# Molecular characterization of hemoglobin D Punjab traits and clinical-hematological profile of the patients

**DOI:** 10.1590/S1516-31802012000400008

**Published:** 2012-09-04

**Authors:** Sanjay Pandey, Rahasya Mani Mishra, Sweta Pandey, Vineet Shah, Renu Saxena

**Affiliations:** I MSc. Senior Research Fellow, Department of Hematology, All India Institute of Medical Sciences, New Delhi, India.; II PhD. Professor, Department of Environmental Biology, Awadhesh Pratap Singh University, Rewa, Madhya Pradesh, India; III MSc. Student, Department of Hematology, All India Institute of Medical Sciences, New Delhi, India.; IV MSc. Senior Research Fellow, Department of Cardiac Biochemistry, All India Institute of Medical Sciences, New Delhi, India.; V MD. Professor, Department of Hematology, All India Institute of Medical Sciences, New Delhi, India.

**Keywords:** Hemoglobinopathies, Hemoglobins, Chromatography, high pressure liquid, Thalassemia, Polymerase chain reaction, Hemoglobinopatias, Hemoglobinas, Cromatografia líquida de alta pressão, Talassemia, Reação em cadeia de polimerase

## Abstract

**CONTEXT AND OBJECTIVE:**

Hemoglobin (Hb) D hemoglobinopathies are widespread diseases in northwestern India and usually present with mild hemolytic anemia and mild to moderate splenomegaly. The heterozygous form of Hb D is clinically silent, but coinheritance of Hb D with Hb S or beta-thalassemia produces clinically significant conditions like thalassemia intermedia of moderate severity. Under heterozygous conditions with coinheritance of alpha and beta-thalassemia, patients show a degree of clinical variability. Thus, our aim was to molecularly characterize the Hb D trait among individuals who were clinically symptomatic because of co-inheritance of alpha deletions or any beta-globin gene mutations.

**DESIGN AND SETTING:**

This was a cross-sectional study conducted in an autonomous tertiary-care hospital.

**METHODS:**

Complete blood count and red cell indices were measured using an automated cell analyzer. Quantitative assessment of hemoglobin Hb F, Hb A, Hb A2 and Hb D was performed by means of high performance liquid chromatography (HPLC). DNA extraction was done using the phenol-chloroform method. Molecular analyses on common alpha deletions and common beta mutations were done using the Gap polymerase chain reaction and Amplification Refractory Mutation System, respectively.

**RESULTS:**

We evaluated 30 patients and found clinical variation in the behavior of Hb D traits. In six patients, the Hb D traits were clinically symptomatic and behaved like those of thalassemia intermedia. Molecular characterization showed that three out of these six were IVS-1-5 positive.

**CONCLUSIONS:**

HPLC may not be the gold standard for diagnosing symptomatic Hb D Punjab traits. Hence, standard confirmation should include molecular studies.

## RESUMO

**CONTEXTO E OBJETIVO:**

Hemoglobinopatias da hemoglobina (Hb) D são doenças amplamente disseminadas no noroeste da Índia e geralmente se apresentam com anemia hemolítica leve e esplenomegalia leve a moderada. A forma heterozigótica de Hb D é clinicamente silenciosa, mas co-herança de Hb D com Hb S ou beta-talassemia produzem condições clinicamente significativas, como talassemia intermediária de gravidade moderada. Em condição heterozigótica com co-herança de alfa e beta-talassemia, pacientes mostram variabilidade clínica. Assim, nosso objetivo foi a caracterização molecular dos traços da Hb D em individuos clinicamente sintomáticos, devido à co-herança de deleções de alfa ou quaisquer mutações gênicas de beta-globina.

**TIPO DE ESTUDO E LOCAL:**

Estudo transversal; realizado em um hospital de cuidado terciário autônomo.

**MÉTODOS:**

Hemograma completo e índices de células vermelhas foram medidos pelo analisador automatizado de células. Avaliação quantitativa de hemoglobina Hb F, Hb A, Hb A2 e Hb D foi realizada por cromatografia líquida de alta eficiência. Extração de DNA foi feita pelo método de fenol-clorofórmio. Estudo molecular para deleções comuns de alfa e mutações comuns de beta foi feito por Gap-reação em cadeia da polimerase e amplificação refratária de mutação, respectivamente.

**RESULTADOS:**

Avaliamos 30 pacientes e verificamos variação clínica no comportamento dos traços da Hb D. Em seis pacientes, os traços da Hb D foram clinicamente sintomáticos e se comportavam como os de talassemia intermédia. A caracterização molecular mostrou que três desses seis pacientes eram IVS-1-5 positivos.

**CONCLUSÕES:**

HPLC pode não ser o padrão ouro para o diagnóstico de traços de Hb D Punjab sintomáticos. Assim, a confirmação padrão ouro deve incluir estudos moleculares.

## INTRODUCTION

Hemoglobin (Hb) D Punjab, also known as Hb D Los Angeles, is an abnormal type of Hb with an amino acid substitution of glutamine for glutamic acid at codon 121 of the beta-globin gene. Hb D occurs in four forms: heterozygous Hb D trait, Hb D-thalassemia, Hb SD disease and the rare homozygous Hb D disease, which usually presents as mild hemolytic anemia and mild to moderate splenomegaly.^1^^,^^2^ Hb D Punjab is one of the most commonly observed abnormal hemoglobin variants worldwide, not only in the Punjab region of northwestern India, but also in Italy, Belgium, Austria and Turkey.^3^^,^^4^^,^^5^^,^^6^^,^^7^^,^^8^ There are a number of reports of Hb D Punjab cases from different regions of Turkey, including Denizli province, in the Aegean region.^2^^,^^7^^,^^9^^,^^10^^,^^11^^,^^12^^,^^13^ Its incidence has been reported by different researchers throughout Turkey with an overall frequency of 0.2%.^13^ In Denizli province, the most common abnormal variant is Hb D Punjab, accounting for 57.8% of the total abnormal Hb S observed in premarital screening.^7^ The Hb D Punjab frequency in Denizli province is similar to the frequency observed in Xinjiang Province, People’s Republic of China, where it accounts for 55.6% of total Hb variants.^14^ Hb D Punjab occurs with greatest prevalence (2%) among Sikhs in Punjab, India, whereas the reported prevalence rate in Gujarat province, in western India, is 1%.^1^ Although Hb D is not uncommon in India, its homozygous form is very rare.^1^^,^^2^^,^^15^

## OBJECTIVE

The aims of this study were to molecularly characterize symptomatic Hb D traits and make a comparative analysis on the clinical-hematological data of such patients.

## MATERIAL AND METHOD

This was a cross-sectional study since the data were obtained from patient records rather than prospectively. The subjects recruited for this study presented Hb D Punjab traits. The duration of the sample collection was three years and the study was conducted in the Department of Hematology, All India Institute of Medical Sciences (AIIMS), in New Delhi. Cases of Hb D traits that were diagnosed by means of high performance liquid chromatography (HPLC) were included, whereas patients with sickle cell disease, homozygous Hb D and Hb D with thalassemia, and patients with other hemoglobinopathies were excluded from the study. Blood samples of around 5 ml were collected from the patients after they had signed an informed consent form. Thirty patients, comprising 16 males and 14 females with a median age of 20 years (range 1-43), were included in the study.

Complete blood count and red cell indices were measured by means of an automated cell analyzer (SYSMEX K-4500, Kobe, Japan). Giemsa-stained peripheral blood smears were examined for red cell morphology. Quantitative assessment of hemoglobin Hb F, Hb A, Hb A2 and Hb D was performed using HPLC (Bio-Rad-Variant, Bio Rad, California, United States). DNA extraction was done by means of phenol-chloroform methods, for reasons of cost-effectiveness and availability. Molecular analyses for common alpha deletions and common beta mutations were done in accordance with descriptions in published studies in the literature.^16^^,^^17^^,^^18^^,^^19^ Ethidium bromide (5 ml) mixed in 2.5% agarose gel and bromophenol blue loading dye was used to detect the mutation in horizontal gel electrophoresis. Mean values, standard deviation and frequency distribution were used to evaluate the hematological and clinical data.

## RESULT

**Figure 1.**
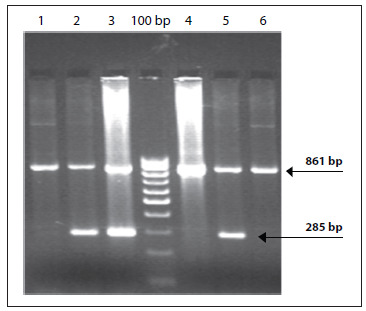
PCR result from six symptomatic patients, in which lanes 2, 3 and 5 are positive for IVS 1-5 mutation (bp = base pairs).

Six out of the 30 patients were clinically symptomatic and presented anemia, jaundice, pallor and weaknesses. The patients’ peripheral smears showed microcytic hypochromic red cells with few target cells. A mild degree of anisopoikilocytosis was noticed. Six Hb D patients presented Hb A2 ranges of 4% to 6.5%, with low mean Hb (8.3 ± 3.0 g/dl), serum iron (35.2 ± 3.4 µg/dl) and red cell indices. Their hematological features included Hb F (normal range < 1.5%) and Hb A2 (normal range < 3.6%). The levels were within the normal range in the remaining 24 patients, and all were asymptomatic. None of the patients had alpha deletions. Out of the six symptomatic patients, three patients were IVS1-5 positive (Figure 1). All the 24 asymptomatic Hb D patients were negative for beta thalassemia mutations. Details of the hematological profile are given in Table 1.

**Table 1.** Comparative hematological profile

Mean ? standard deviation		
Hematological parameters	Symptomatic Hb D n = 6	Asymptomatic Hb D n = 24
Hb A0	48.6 ? 3.2	51.2 ? 8.9
Hb A2	4.2 ? 1.8	2 .2 ? 1.7
Hb F	2.5 ? 1.3	1.2 ? 0.4
Hb D	42.6 ? 3.5	39.0 ? 10.8
Red blood cells (millions/μl)	3.2 ? 1.7	4.6 ? 1.0
Hemoglobin (g/dl)	8.3 ? 3.0	11.4 ? 3.2
Hematocrit (%)	27.2 ? 2.6	30.8 ? 7.9
Mean corpuscular volume (fl)	72.3 ? 5.4	74.5 ? 11.2
Mean corpuscular hemoglobin (pg)	22.7 ? 2.3	24.9 ? 4.9
Mean corpuscular hemoglobin concentration (g/dl)	25.3 ? 2.4	28.2 ? 3.5
Serum iron (µg/dl)	35.2 ? 3.4	75.6 ? 8.3

Hb = hemoglobin.

## DISCUSSION

Homozygous Hb D disease is rare and usually presents with mild hemolytic anemia and mild to moderate splenomegaly. Heterozygous Hb D is a clinically silent condition, but coinheritance of Hb D with Hb S or beta thalassemia produces clinically significant conditions like sickle cell anemia and chronic hemolytic anemia of moderate severity. Although Hb D is not uncommon in India, its homozygous form is very rare^1^^,^^2^^,^^15^ and very few case reports have been reported.^20^ The major concern in ruling out Hb D beta-zero thalassemia is that homozygous Hb D disease causes mild hemolytic anemia, but coinheritance of beta-zero thalassemia seems to produce deleterious effects relating to the presentation of Hb D disease, thus leading to chronic hemolytic anemia of moderate severity.^21^ An association between Hb D and hematological malignancies has also been reported.^22^ Earlier studies from Pakistan, Iran, United Arab Emirates and Mexico have shown that the clinical presentation of Hb SD disease cases is similar to that of patients with the severe form of sickle cell anemia.^23^^,^^24^ On the other hand, reports from India have shown variable clinical manifestations of Hb SD disease.^25^^,^^26^

There are no detailed reports on the clinical and hematological profile of Hb D patients, and only a few case reports have been published.^20^ In our sample, six patients with Hb D traits were clinically symptomatic and the disease behavior was like cases of thalassemia intermedia. However, the red cell indices were low, which may have been due to coinheritance of either alpha deletion or beta mutation. Therefore, it is necessary to conduct molecular analyses for alpha and beta mutations in these cases, in order to ascertain the factors causing modulation of disease severity. The molecular diagnosis showed that three out of these six cases were IVS1-5 positive. The other three clinically symptomatic cases may have been caused by some other disease that had not been characterized. Factors such as environmental influence, genetic polymorphism in the beta-globin gene clusters, expression of fetal hemoglobin and coinheritance of alpha thalassemia may contribute towards the diversity of disease conditions. A report from Saudi Arabia has also emphasized the importance of careful analysis of the electrophoresis results and the usefulness of molecular studies in premarital screening and other hemoglobinopathy screening programs.^27^ A study in Spain concluded that the hematological picture revealed that Hb D Punjab was a mild condition, but that the factor reliably responsible for the phenotype was an imbalance in globin chain synthesis, because of frame-shift CD 8/9 (+ G) beta-zero thalassemia mutation.^28^ A hematological and molecular report on Hb D Iran associated with beta-zero thalassemia (619 base-pair deletion) mutations showed a hypochromic, microcytic red cell picture with reduced red cell indices.^29^ Only a few case reports are available in Hb D hemoglobinopathies. None of these investigations were on large populations of Hb D hemoglobinopathies. Therefore, controversy still exists with regard to the clinical features of Hb D coinheritance with either alpha deletions or beta mutations. However, IVS 1-5 (G→C) is the commonest beta globin gene mutation in India^30^ and has a severe effect on the clinical phenotype of thalassemia and sickle beta thalassemia patients.

## CONCLUSIONS

The diversified nature of Hb D Punjab traits is due to interaction of other factors that act epistatically on the clinical severity of the disease, given that the HPLC results are contradictory with the clinical findings. Thus, it is strongly recommended that molecular studies should be conducted.
